# Lonely, harassed and abandoned in society: the lived experiences of Iranian homeless youth

**DOI:** 10.1186/s40359-022-00787-8

**Published:** 2022-03-20

**Authors:** Fatemeh Johari, Abedin Iranpour, Mahlagha Dehghan, Somayeh Alizadeh, Mansoure Safizadeh, Hamid Sharifi

**Affiliations:** 1 HIV/STI Surveillance Research Center, and WHO Collaborating Center for HIV Surveillance, Institute for Futures Studies in Health, Kerman University of Medical Sciences, Kerman, Iran; 2 Nursing Research Center, Kerman University of Medical Sciences, Kerman, Iran; 3 Social Determinants of Health Research Center, Institute for Futures Studies in Health, Kerman University of Medical Sciences, Kerman, Iran

**Keywords:** Homeless youth, Loneliness, Social isolation, Adolescent

## Abstract

**Introduction:**

Homelessness is increasing among young adults in large cities. According to the United Nations, there are more than one billion absolute or relative homeless people in the world. This study was conducted to explain the lived experiences of homeless youth in southeastern Iran.

**Materials and methods:**

In this qualitative study, we recruited 13 participants in a big city, southeast Iran, in 2020. The participant was young homeless adults aged 18–29 years who were using homeless shelters provided by the municipality, sleeping in parks or on streets. Data were collected through in-depth and semi-structured interviews and three focus group discussions. Data were analyzed by conventional qualitative content analysis.

**Results:**

The main category of “lonely, annoyed, and abandoned in society” and three subcategories of avoidance of/by society, comprehensive harassment, and lack of comprehensive support were extracted. The experiences of young homeless adults showed that they escaped from the community due to addiction, feeling like a burden to others, and social isolation, and not only have they been left without support in society, but they have also suffered from all kinds of physical and psychological harassments.

**Conclusion:**

The lived experiences of homeless people show that in addition to appropriate facilities and living conditions, they require respect, reduced social stigma, discrimination, and favorable conditions for a return to life. Therefore, authorities should identify and settle their problems and needs.

## Introduction

Homelessness has become a social problem due to the growing population and lack of welfare facilities in many countries of the world [1, 2]. Homelessness is an unpleasant experience in large cities and predisposes to discrimination, rejection, and many hardships for people [3].

Marginalization and homelessness are associated with social rejection, and living in a cardboard box is the worst consequence of rejection among the homeless [4]. Poverty and homelessness are on the rise in cities where marginalization is growing exponentially, and practical measures have rarely been taken to tackle homelessness in the world. Preliminary studies on the cycle of homelessness, incorrect understanding of the cause of homelessness, ignorance of the housing issue, lack of recognition of homelessness, lack of resources, and lack of legal enforcement are factors that make combating against homelessness unsuccessful [5].

United Nations Human Settlement Program reports that there are more than one billion homeless people in the world [6].There are no accurate statistics on this population in Iran due to lack of large-scale survey, lack of a single organization to organize the homeless, and the parallel work of governmental and non-governmental (NGO) agencies [7]. According to the report of Tehran Municipality in 2015, 20,000 homeless people were using the shelters provided by the municipality. This information indicates that homelessness is growing in Tehran. Homeless shelters also included overnight accommodation, social and health services, and keeping them away from the city, but few rehabilitation services [3].

The United Nations defines homelessness as “absolute and relative homelessness.” Absolute homelessness refers to people living on the street, vehicles, and abandoned uninhabitable buildings. Relative homelessness refers to people living in spaces that do not meet the basic health and safety standards, including protection against the elements, access to safe water, personal safety, and reasonable price [1].

Family conflict is the biggest problem facing young people, which has led to their homelessness. In addition, physical abuse and emotional and sexual harassment are often considered reasons for their homelessness [8, 9]. Substance abuse (SA), mental health problems, family breakdown, and housing are other reasons for this problem [10]. Many homeless people enter the cycle of SA, and alcohol and SA commonly perpetuate homelessness [11]. Numerous factors also contribute to the persistence of homelessness, including a set of individuals, economic, social, environmental, and family characteristics [3]. Research has also shown that an increase in the family relationship and financial and emotional support reduces the length of homelessness, and employment is an essential factor in shortening the period of homelessness [3].

Most homeless people have numerous health problems, including malnutrition, anemia, infectious and respiratory diseases, mental illness, diarrhea, oral and dental diseases, and reproductive system infections [12–14]. The incidence of asthma, tuberculosis, influenza, pneumonia, hepatitis, lice, and scabies increases among young homeless adults. The rate of tuberculosis among the homeless is 20 times higher than that of the general population [8, 13]. The rate of sexual abuse has also been 35% [13]. The US National Homelessness Survey showed that 30–60 percent of young homeless women experienced unwanted pregnancies. These people had to choose sex for food, money, or clothes because of limited income and livelihood, and they often did not use contraceptives [10].

The most meaningful experiences of homeless people reported: rejection, trauma, social isolation, lack of educational-welfare infrastructure, negative capital, and social indifference [11]. Also, the results of one qualitative study showed that sleeping in cardboard boxes depended on macro, structural and individual factors. The social conditions of these people should be changed, and people sleeping in cardboard boxes can effectively change this condition [6]. The health care of homeless and vulnerable people did not meet international standards of patient care [15].

Homelessness is an unpleasant and growing experience that involves more young adults every day. Limited studies are available in this regard due to the neglect of the problems of this group. Therefore, we decided to explain their experiences to recognize better and control this social damage and plan for their rehabilitation. This study aimed to explain the lived experiences of young homeless adults aged 18–29 years in southeast Iran.

## Materials and methods

### Study type and setting

In this qualitative study, 13 homeless participants in a big city in southeast Iran were recruited using convenience sampling. Data were collected using individual interviews. Individual interviews were done in a space away from other people in the park or inside the car of the first author (FJ). The focus group discussion also took place in a quiet room where participants could feel free, and we could record their voices. The interviews with men were conducted at a comprehensive health center and a room at the Women's Behavioral Diseases Counseling Center near their gathering place.

### Participants and data collection

This study was performed on young absolute homeless adults aged 18–29 who were sleeping in homeless shelters, parks, and streets from July 2019 to November 2020. Inclusion criteria included young adults living in one of the main squares of the studied city or homeless shelters. Most participants were male, single, and had primary education in the present study. Most of the participants in the study (n = 5) had been homeless for less than a year. Table 1 shows the demographic characteristics of the participants. The study was done at night when charities distributed food among the homeless people in the squares. After acquiring informed consent and providing material rewards, this study was conducted by semi-structured interviews with the homeless to examine the experiences of these people by themselves. Exclusion criteria were the dissatisfaction of these people to participate in the interview.

**Table 1:** Demographic characteristics of the participants

Participants number	Age	Sex	Marital status	Length of living in cardboard box	Education level
1	38	Female	Widow/er	< one year	Uneducated
2	28	Male	Married	One-two years	Uneducated
3	30	Female	Single	> four years	Diploma
4	32	Male	Single	One-two years	Diploma
5	29	Female	Widow/er	< one year	Uneducated
6	30	Male	Married	< one year	Diploma
7	32	Male	Married	> four years	Elementary
8	35	Male	Single	> four years	Uneducated
9	26	Male	Single	Two-four years	Elementary
10	30	Female	Divorced	< one year	Elementary
11	36	Male	Divorced	Two-four years	Elementary
12	29	Female	Divorced	One-two years	Diploma
13	35	Male	Divorced	< one year	Elementary

Thirteen in-depth and semi-structured interviews and three focus group discussions were used as the main data collection methods. Such interviews are suitable for qualitative research due to their depth and flexibility. At the beginning of each interview, the characteristics of each sample, including age, sex, marital status, and education, were recorded. The first author conducted and recorded all individual interviews. Interviews lasted between 2 and 60 min, depending on the participants. The interview guide consisted of several key questions based on which the participants shared their experiences. According to the answers received, additional questions were asked to find out the different parts of the subject. The questions were as follows: would you please describe your experiences of homelessness? Did you experience any problems and challenges? How did you solve them? How did the authorities and society help you? The probing questions were, “please explain more? What do you mean? Why and how? Can you give an example?”.

Due to the particular experience of these people, we felt during the individual interviews that some issues might be better discussed in focus group discussions. Then three focus group discussions were conducted with the same people who participated in the individual interviews [16, 17]. The samples were also divided into two groups of four men and one group of five women. In each focus group, the second author conducted the interviews, and the first author took notes to clarify the material better and prevent the loss of nonverbal information. The same semi-structured interviews were used. Each session lasted 45–60 min. These interviews were also recorded and transcript verbatim.

### Data analysis

All individual interviews and focused group discussions were analyzed by Graneheim and Lundman conventional qualitative content analysis [18]. The first author transcribed the recorded interviews verbatim and repeatedly read the data obtained from the interviews to get a general perception, according to Graneheim and Lundman. First, we determined the unit of analysis. In this study, each interview was considered as a unit of analysis. Second, the text was divided into meaning units. Each meaning unit consists of words, sentences, or paragraphs containing aspects related to each other through their content and context. Third, we condensed the meaning units while still preserving the core. Fourth, the condensed meaning units were labeled with a code, and sub-categories were created. Fifth, we created the categories that were the core feature of qualitative content analysis. In addition, we used nonverbal information to understand the phenomenon and better code the data. The first author was responsible for transcription and initial coding, and the other authors were responsible for the critical review of all steps.

Interviews with young homeless adults in the first phase resulted in the extraction of 264 codes. The codes were refined to 157 after several meetings and discussions with the research team. These codes were classified into 11 subcategories, three categories, and one theme (Table 2).

**Table 2:** Classification of the homeless youth experiences

Theme	Categories	Sub-categories
Lonely, harassed, and abandoned in society	Social isolation	Feeling like a burden
		Social isolation
	Comprehensive harassment	Social harassment
		Physical abuse
		Sexual harassment
		Psychological harassment
		Financial harassment
	Lack of comprehensive support	Lack of family support
		Lack of support to meet basic needs
		Lack of access to health facilities and medical support
		Inadequate charitable donations

### Trustworthiness

Qualitative research typically uses four topics to describe different aspects of trustworthiness: credibility, confirmability, dependability, and transferability [19]. Several strategies were applied in the current study to increase trustworthiness. By remaining in the field for a long time (one year) to collect and evaluate data in the present study, the researcher aimed to build a good relationship with the participants. The researcher attempted to pick volunteers with various qualities to acquire more in-depth data (maximum variation). After reviewing each interview, we referred back to the participants and made adjustments as needed to clarify ambiguities and corroborate the extracted opinions (member check). In addition, two professional researchers were asked to examine and interpret the data, and all extracted codes and categories were verified and approved by the authors to ensure credibility. The study team created and printed a mind map during the research process to improve data confirmability. Two research team members (qualitative research experts) were provided the transcripts of multiple interviews and the codes and extracted categories to double-check the data coding process's accuracy. To establish dependability, the external observer strategy was used in this study to examine his likely comparable knowledge with the researcher and look for contradicting situations. As a result, the data were given to two researchers (both of whom were experts in qualitative research) who affirmed the data's reliability based on the same concept. The research findings were given to two non-participant samples from the current study to improve the data's transferability and appropriateness. Their comments were solicited, with a conceptual generalization based on the similarities. We have made an effort to detail all of the research steps.

## Findings

### Lonely, harassed and abandoned in society

According to the experiences of young homeless adults, they have escaped from the community due to SA and addiction, feeling like a burden to others and social isolation, and not only have they been left without support in society, but have also suffered from all kinds of physical and psychological harassments. The main category extracted from their experiences was “lonely, annoyed and abandoned in society.” The main category consists of three secondary categories: avoidance of/by society, comprehensive harassment, and lack of comprehensive support.

### Avoidance of/by society

This category includes feeling like a burden to others and social isolation. Most homeless people suffered from avoidance of/by society due to poor appearance, poor health, SA and addiction, unemployment, and tensions with their families and friends. Sometimes they blame themselves or others for the unhealthy conditions of their lives. Feeling like a burden to family and community, they always try to live alone and away from the district. These people leave their homes due to easy access to narcotic drugs and keep themselves away from any pressure and discomfort. A woman described her experience of feeling like a burden to others as follows: “*My sister's husband kept shouting at my sister and complaint about the presence of me at his house, so I left the house.*” (Participant No. 3).

All homeless people rejected by their families have such a feeling, and they are sometimes unaware of their families for years, and no one pays attention to them. These people are also isolated due to a loss of self-confidence and social trust. Participant No.4 described his life as follows: “*I have nothing to do with anyone, and I am alone.*”

Another man, who has been homeless for three years, stated his experience as follows: “*I have been rejected because of addiction, I have a family, but I am ashamed of my behavior, and my family would not like to see me.*”

### Comprehensive harassment

This category includes social harassment, physical abuse, sexual harassment, psychological harassment, and financial harassment.

#### Social harassment

One of the problems of homeless people was their messy appearance and poor health. In addition, other people always disrespect and neglect them because they have to behave immorally to earn a living. They are always stigmatized and discriminated by society.

“The staff working in this center sometimes show disrespectful behaviors, which make us upset and anxious.” (Participant No. 4).

“We are addicted, so if somebody lost something, we would be blamed for them, and nobody would believe us.” (The second group discussion).

#### Physical abuse

Homeless people have always been affected by various forms of harassment and problems due to lack of permanent shelter, lack of money, and living on the streets. These problems include a victim of street fights, car accidents, and various illnesses. In addition, these people cannot withstand these injuries due to lack of proper nutrition, a severe addiction to multiple substances, and poor physical condition.

“A group of people consume alcohol at nights and annoy the unfortunate addicts, and the police do not pay attention to us.” (Participant No. 9).

#### Sexual harassment

Sexual harassment was common among women that had caused fear and insecurity among them. This group of people, especially women, are the most defenseless and have realized many problems. One female participant, who was abused sexually, described her experiences as follows:

“When we are sleeping in parks, the drunk boys annoy us, and we can do nothing, we are threatened to be stabbed by them, so we have to let them do whatever they want and rape us.” (Participant No. 12).

#### Psychological harassment

Homeless people have experienced hardships and problems such as family rejection, vagrancy, and all kinds of street harassment throughout their lives. These factors have made them very psychologically vulnerable, and the existing anxiety and stress have deprived them of peace of mind.

“At night, a group us harass the miserable addicts.” (Participant No. 9).

#### Financial harassment

Lack of financial security and loss of all property are problems facing homeless people living on the streets. Their friends or people like them often grab their belongings.

“We are not secure when we are sleeping in the park at night. When we wake up in the morning, we will see that our friends have grabbed all our property.” (Participant No. 12).

### Lack of comprehensive support

This category contains four subcategories: lack of family support, lack of support to meet basic needs, lack of access to health facilities and medical support, and insufficient charitable support.

#### Lack of family support

Homeless people are unwilling to return home because of a lack of emotional family support and family problems. Most homeless people preferred to live on the streets because of the lack of daily worries, loneliness, and family disputes and conflicts.

A woman described her experience as follows:

“When my parents died, my uncle kicked me out of the parental house; my siblings are married and have nothing to do with me.” (Participant No. 12).

“I had to leave my house because nobody paid attention to me.” (Participant No. 2).

#### Lack of support to meet basic needs

Adequate shelter, job, and adequate access to food are among the basic needs of these people. The majority of the participants have no shelter and live on the streets. Annoying noises such as the sound of vehicles and night fights also discomfort them. They have more problems finding a place to sleep, especially in the cold season, so they are more willing to use homeless shelters. They also believe that sleeping in the parks has made the city look unattractive. A man participating in the first group discussion described his experience as follows:

“Homeless shelters have been closed, and we do not know what to do during rainfall, now we need a place to sleep, they used to give us a bed, we used to consume drugs there and took a shower. Some of these homeless shelters have been closed or relocated due to complaints of neighbors, which have made it difficult for us to access them.”

The majority of the participants also considered unemployment the main cause of their addiction and homelessness, and many wished to find a suitable job. These people cannot find suitable jobs due to their messy appearance, addiction, distrust of employers, so they become waste pickers and beggars to earn a living. The experience of interviewee No. 7 was as follows:

“If addicted people quitted addiction, they would return to it because they are unemployed.”

The majority of the homeless people we interviewed used their money for narcotic drugs. They always met their nutritional needs by salvaging reusable or recyclable materials and preparing junk, harmful food, and snacks. As a result, they suffered from malnutrition and digestive problems. Non-governmental organizations sometimes prepare food for these people, the quality of which should be checked, and if possible, they should be designed purposefully and according to nutritional standards.

#### Lack of access to health facilities and medical support

Were other experiences of the young homeless adults. These people have realized the poor level of health facilities due to the lack of permanent shelter and living on the streets. One of the main shortcomings of these people was inaccessibility to a suitable place for bathing, sanitary services, and detergents. Homeless women had more challenges in accessing health services such as sanitary pads. They had to use inappropriate methods and tools due to economic problems and a lack of support from the authorities. One of the problems of these people in the field of medical facilities was the lack of money and the high cost of medical services, so they preferred self-medication.

“I do not have enough money to spend for sanitary pads every month. I wash napkins and use them as pads, or I sometimes use tissue papers.” (Participant No. 10).

“Sometimes we get sick, but God helps us because we do not have anybody. Our pain gets better quickly, and we do not visit the doctor, and our illness will be treated with herbal medicines or those prepared from a pharmacy.” (Participant No. 13).

#### Inadequate charitable donations

Most homeless people depend on charitable donations to provide daily food, seasonal clothing, health supplies, and free medical care. The lack of this support has made it difficult to meet their needs.

“Once every 5–6 days, they come from the camp and give us little equipment.” (Participant No. 2).

## Discussion

This study was conducted to explain the experiences of homeless people. The analysis of the interviews showed that the young homeless adults suffered from a wide range of problems, and the relevant organizations always ignored them. Lonely, annoyed, and abandoned in society were defined as the lived experience of homeless youth. This theme includes three categories of avoidance of/by society, comprehensive harassment, and lack of comprehensive support.

Avoidance of/by society: According to the participants’ experiences, feeling like a burden to others and social isolation are among the lived experiences of homeless people. The participants in this study considered their existence as a burden to those around them because family and society neglected them. Existing evidence shows that addiction, physical and mental disabilities, and no adaptation to the environment are among the factors facilitating homelessness and paving the way for society's avoidance [20]. In addition, the rate of avoidance to the community is very high in homeless people, especially in substance-dependent homeless women [21]. Other studies consider social isolation, rejection, and social indifference as lived experiences of homeless people [11]. Return of the homeless people to their homes can be facilitated by reducing stigma and discrimination, increasing a sense of respect and social support. In addition, social facilities such as a job that connect them to society should be provided to make them sociable.

Comprehensive harassment: Homeless people in the present study have realized many problems due to different lifestyles from the public, including various dimensions of insecurity and various social, sexual, physical, psychological, and financial harassment. They often face difficulties for their survival, and sometimes they have to endure conditions beyond their human dignity and undermine their self-esteem. The results of studies show a decrease in life expectancy and early death of homeless women with feelings of failure and inferiority, lack of hope to return to normal conditions, and the desire to die [22]. The use of HIV prevention methods reduces stigmatization and disrespect and promotes self-confidence [23]. The incidence of physical harassment and the high rate of trauma in these people have always been discussed. Findings of studies also show that substance abuse, sexually transmitted infections, childhood traumas, and gender are significantly associated with sexual assault among women and different types of injury and victimization in men [11, 24, 25]. Evidence also showed a high prevalence of drug abuse and another gender status among young people with a history of sexual abuse. Early sexual abuse affected street drug abuse through getaway behavior [24, 26]. This group of people experienced a high level of psychological harassment. O’Brien et al. obtained similar results when examining mental health outcomes among homeless youth. They believed that homeless youth had poorer mental health outcomes than their peers living in a shelter [27]. Loss of property and belongings while sleeping or using drugs is another dimension of insecurity that a similar study was not found to compare.

Lack of comprehensive support: Family and community inattention and lack of support was another experience of homeless people in the present study. Most of them became homeless due to family tensions caused by addiction, financial problems, and incompatibility of family members. The participants in this study cannot defend their social rights due to their inability to communicate appropriately. The negligence of the relevant authorities or the insults and ridicule of those around them have refrained them from using and receiving social services such as health services. The studies also showed a significant difference between the homeless and non-homeless people in emotional support. Homeless people receive less emotional support than non-homeless ones [1, 28]. Homeless people do not refer to health centers due to the inappropriate behavior of some personnel. Their different sleeping and waking hours from service hours are another reason for not using services. This finding confirms the results of previous studies, which found that homeless people had poorer health status than the general population [15, 29, 30]. Lack of insurance, expensive services, and limited services for this group are why they do not refer to receive medical services. The results also show that homeless people face significant barriers such as perceived structural (limited clinics, limited hours of activity, prioritized health conditions, and long waiting times) and social barriers (discrimination perceived by indifferent professionals, executives, and society) that impede their access to health care services [8, 25, 31]. Homeless people have always perceived many problems and experiences and have become part of today's society. The irresponsibility of governmental organizations and institutions has made charitable institutions important. These institutions can play an important role in improving the living conditions of the homeless, but these people experienced many limitations due to the lack of charitable donations. No similar study was found in this regard.

Homeless people have problems accessing everyday basic needs such as shelter, proper jobs, and adequate food. It is essential to have a good place to sleep in all seasons of the year, find a job, and a source of income to access food resources. However, this finding contradicted the results of Fathi et al. regarding the need of homeless youth for a suitable place to sleep. They described the preservation of a distinct identity and the inefficiency of homeless shelters as the main reasons homeless people were not using homeless shelters in Tehran [32]. This finding was consistent with the results of other studies. The shelter is important in preventing death and illness on the street, motivating them to return to the community, increasing life expectancy, and social participation among clients [2]. The role of adequate shelter is vital in preventing homelessness, but financial support and employment are important factors in shortening the period of homelessness [1, 3, 6, 26]. However, limitations in this area have made homeless people become waste pickers and beggars. They also find their food by salvaging reusable materials, which causes malnutrition and various diseases. Studies have shown that the purchase rate of less healthy food was higher than that of healthy food. Regardless of nutrient density, homelessness reduced the likelihood of purchasing most nutritional food [33].

## Limitations

Difficulty in determining the inclusion criteria was among the problems and limitations in this study. They had no birth certificate or national ID card, so we had to accept their self-declaration. It was also challenging to determine the accurate number of homeless people because of differences in the definition of homelessness. In addition, access to these people was difficult due to the uncertainty of their sleeping place and their dispersion. It was challenging to find them and gain their trust for an interview, and they were willing to cooperate only in case of receiving material incentives. Their different waking hours from the general population was another limitation of the study, so we interviewed them in the evenings for easier access. The majority of interviews were conducted after eight pm. There were limited studies conducted on homeless youth, especially limited qualitative and quantitative studies on the needs of homeless people, so we had limitations in comparing the results with similar studies. One of the most important factors in trustworthiness is how participants are selected. Although we tried selecting participants of different genders and ages with different experiences, the maximum variation may not be completely fulfilled. Other studies with participants with more different characteristics are needed. Transferability is another important factor in the trustworthiness of the study. To facilitate the achievement of this factor, a clear description of the culture and context of the study, the characteristics of the participants, and the process of data collection and analysis is needed. Although we explained the participants' characteristics and the study procedure in detail, the finding of the present study cannot be generalized to other communities, and the finding could be transferred only to communities with a similar culture.

## Conclusion

The results of this qualitative study among homeless people showed that these participants, who are in avoidance of/by society due to their circumstances, have lost all their support and have been subjected to various forms of harassment. In addition to the need for facilities and planning to achieve appropriate living conditions, these people should be respected, less stigmatized, and discriminated against. Favorable conditions should be created for their returns to life. It seems that family conflicts are significant to prevent homelessness of young adults. Therefore, policymakers and executives should identify the problems and needs of homeless youth and take action to address them. It can also direct the activities and services provided for this group currently or in the future and predispose them to quantitative studies. Therefore, researchers can examine the different dimensions of each of the identified cases in different age and sex groups and offer new solutions to solve this social problem. It is suggested that the experiences of homeless people in other populations be examined to further generalize the results in future studies.

## Data Availability

The interviews are in Persian. On request, the written interviews are available from the corresponding author (a.iranpour@kmu.ac.ir).
